# Supplementary material for: TORC2-Dependent Ypk1-Mediated Phosphorylation of Lam2/Ltc4 Disrupts Its Association with the β-Propeller Protein Laf1 at Endoplasmic Reticulum-Plasma Membrane Contact Sites in the Yeast Saccharomyces cerevisiae
Source: Biomolecules. 2020 Nov 25;10(12):1598. doi: 10.3390/biom10121598 (PMC7760575; doi:10.3390/biom10121598)
Supplement: Supplementary file 1 [file biomolecules-10-01598-s001.pdf]

SUPPLEMENTAL MATERIAL

for

TORC2-dependent Ypk1-mediated phosphorylation of Lam2/Ltc4 disrupts its association with the β-propeller protein Laf1 at endoplasmic reticulum-plasma membrane contact sites in the yeast *Saccharomyces cerevisiae*

Magdalena Topolska,[1,2] Françoise M. Roelants,[1] Edward P. Si,[1,3] and Jeremy Thorner[1]*

[1]*Division of Biochemistry, Biophysics and Structural Biology, Department of Molecular and Cell Biology, University of California, Berkeley, CA 94720-3202 USA*

[2]*Present address: Department of Biochemistry and Molecular Biology, Villum Center for Bioanalytical Sciences University of Southern Denmark, 5000 Odense, Denmark*

[3]*Present address: Eastern Virginia Medical School, P.O. Box 1980, Norfolk, VA 23501-1980 USA*

<u>Running Title</u>: Mechanism of inhibition of retrograde sterol transport

*To whom correspondence should be addressed:
Professor Emeritus Jeremy Thorner
Division of Biochemistry, Biophysics and Structural Biology
Department of Molecular and Cell Biology
University of California at Berkeley
Room 526, Barker Hall
Berkeley, CA 94720-3202 USA
Phone: (510) 642-2558
FAX: (510) 642-6420
e-mail: jthorner@berkeley.edu

KEYWORDS: membrane contact sites; ergosterol; regulation; protein kinases; homeostasis

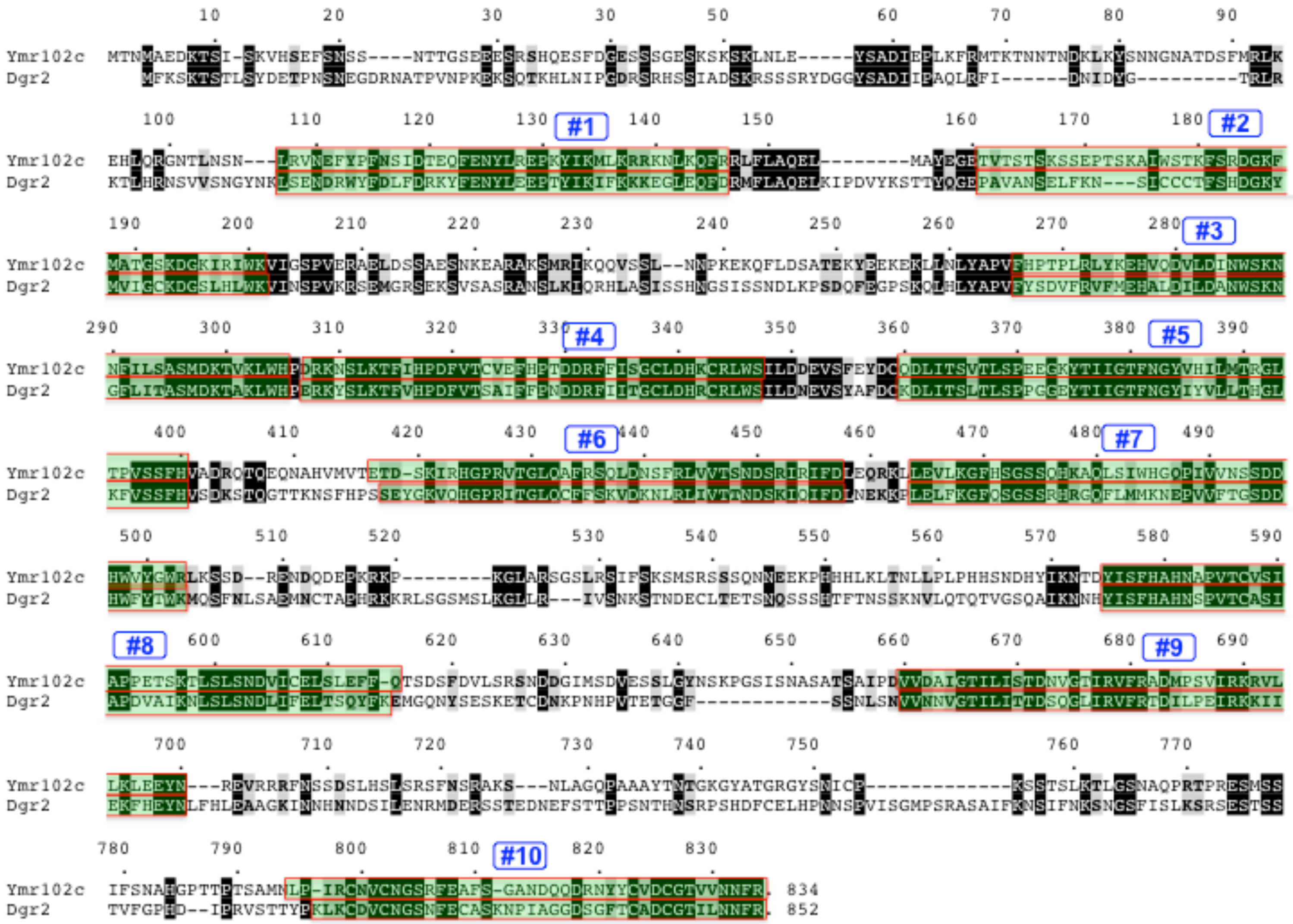

Fig. S1. Laf1 and Dgr2 are predicted ten-bladed β-propeller proteins.

The paralogous *S. cerevisiae* proteins Laf1/Ymr102c and Dgr2/Ykl121w, first identified as components of ER-PM CSs using mass spectrometry (Murley *et al*., 2017), were aligned by NCBI-BLAST (Johnson *et al*., 2008) with identities (white letters on a black box), standard conservative substitutions (bold letters in a gray box), and one-residue gaps (-) to maximize the alignment. The ten apparent WD40 repeats (#1-#10) predicted by the WD40-repeat protein Structure Predictor algorithm (WDSP) (Wang et al., 2013; Wang et. al, 2015), which constitute the ten blades of their predicted β-propeller fold, are indicated and the corresponding sequences highlighted in green shading and boxed in red.

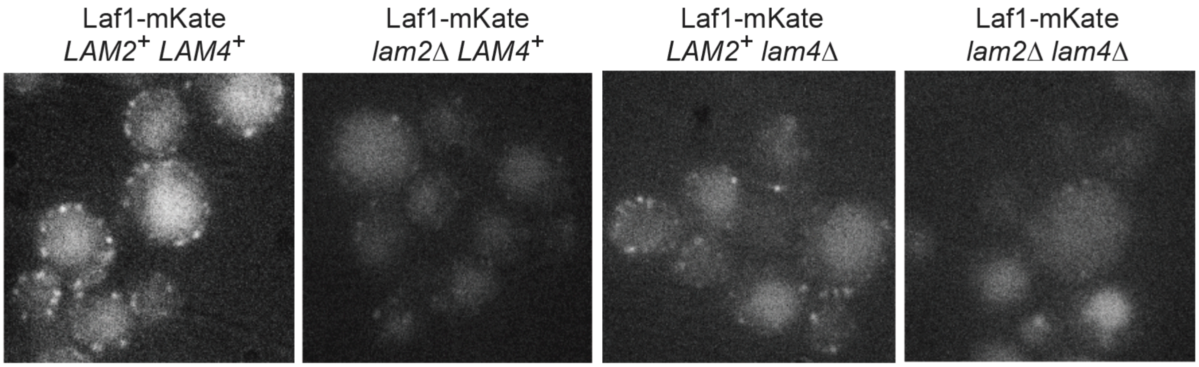

Fig. S2, Panel A. Localization of Laf1 to ER-PM CSs requires Lam2 and Lam4. Same as in Fig. 1C, except that the strains, wild-type (YFR651-A), *lam2*Δ single mutant (YFR652-B), *lam4*Δ single mutant (YFR653-B), or *lam2*Δ *lam4*Δ double mutant (YFR654-B), as indicated, expressed Laf1-mKate (instead of Laf1-mNG).

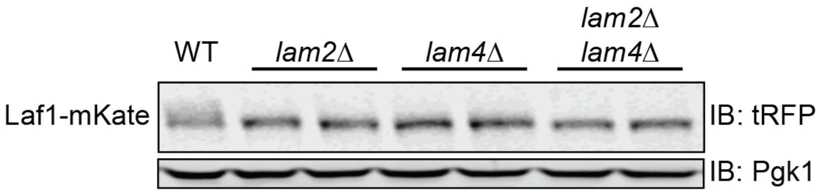

Fig. S2, Panel B. Level of Laf1-mKate unaffected by absence of Lam2 and/or Lam4. Extracts of the same cells as in (A) above were resolved by SDS-PAGE and analyzed by immuno-blotting, as described in Materials and Methods. Note that the level of Laf1-mKate remains stable, but is not phosphorylated, when cells lack Lam2 and/or Lam4.

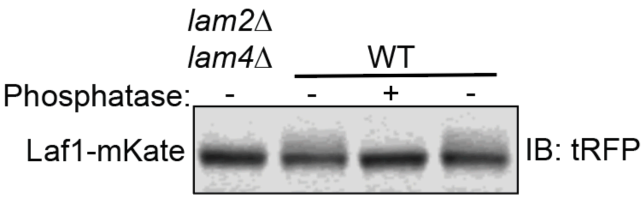

Fig. S2, Panel C. Demonstration that Laf1 is a phospho-protein and only phosphorylated when Lam2 and Lam4 are present. Extracts of either a *lam2*Δ *lam4*Δ double mutant expressing Laf1-mKate (YFR654) or otherwise isogenic WT cells expressing Laf1-mKate (YFR651) were either treated (+) or not (-) with calf intestinal phosphatase, as indicated, and then resolved by SDS-PAGE and analyzed by immuno-blotting, all as described in Materials and Methods.

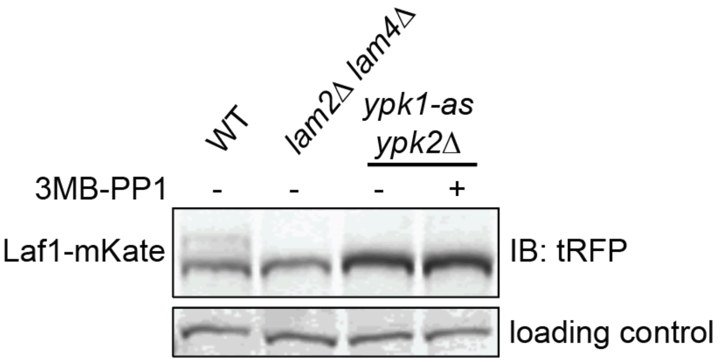

Fig. S2, Panel D. Phosphorylation of Laf1 is dependent on Ypk1 and Ypk2. Extracts of cells of the indicated genotypes, W (YFR651), *lam2*Δ *lam4*Δ (YFR654), and *ypk1-as ypk2*Δ (YFR660), each expressing Laf1-mKate, were grown to mid-exponential phase and then treated with vehicle (DMSO) (-) or with 3MB-PP1 (10 μM final concentration) in the same solvent (+) for 1 h, as indicated, harvested, extracts prepared, and analyzed by SDS-PAGE (6.5% gel) and immunoblotting. (To stimulate TORC2-mediated activation of Ypk1-as, the *ypk1-as ypk2*Δ strain expressing Laf-mKate (YFR660) cells were pre-treated with 1.25 μM myriocin for 2 h before exposure to the Ypk1-as inhibitor 3MB-PP1).

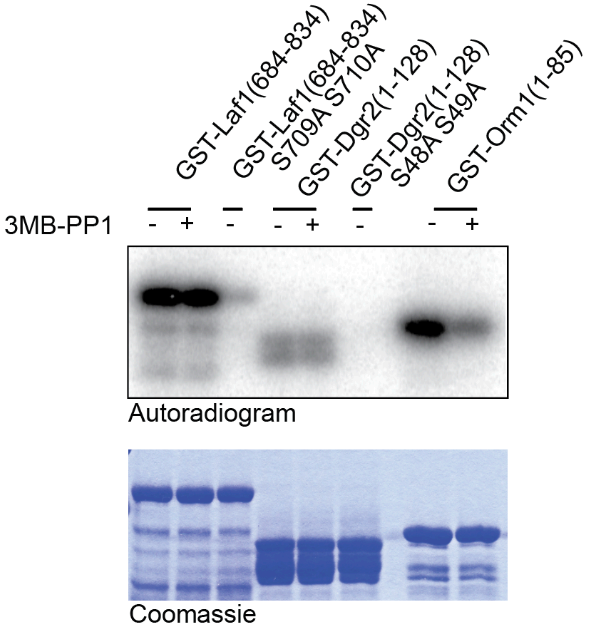

Fig. S2, Panel E. Phosphorylation of Laf1 and Dgr2 occurs at their predicted Ypk1 consensus sites. GST-Laf1(684-834) (pFR398), GST-Laf1(684-834) S709A S710A (pFR402), GST-Dgr2(1-128) (pFR399), GST-Dgr2(1-128) S48A S49A (pFR403), and GST-Orm1(1-85) (pFR203) were purified from *E. coli* and incubated, in the absence (-) or presence (+) of 3MB-PP1, with [γ-^{32}P]ATP and Ypk1-as-His6-HA that was purified from *S. cerevisiae* strain yAM135-A (*ypk1-as ypk2*Δ) containing plasmid BG1805 expressing Ypk1-as-His6-HA-3C-ZZ, as described in detail elsewhere (Muir et al. 2014; Muir, 2015). The products were resolved by SDS-PAGE and analyzed, as indicated. Unlike the control (Orm1), a known Ypk1 substrate (Muir et al., 2014), incorporation into the Laf1 and Dgr2 fragments was not prevented by the Ypk1-as inhibitor 3MB-PP1, even though incorporation was abrogated by Ser-to-Ala mutations at the same sites. Thus,

an as yet unidentified protein kinase in the Ypk1-as-His6-HA preparation is responsible for the observed incorporation into Laf1 and Dgr2 in this *in vitro* context.

REFERENCES

Johnson, M., Zaretskaya, I., Raytselis, Y., Merezhuk, Y., McGinnis, S., and Madden, T.L. (2008) NCBI BLAST: a better web interface. Nucleic Acids Res. 36: W5-9.

Muir, A. (2015) Systematic identification of proteins regulated by the TOR Complex 2-dependent kinase Ypk1 in *Saccharomyces cerevisiae*. Ph.D. Thesis, University of California, Berkeley, 123pp.

Muir, A., Ramachandran, S., Roelants, F. M., Timmons, G. and Thorner, J. (2014) TORC2-dependent protein kinase Ypk1 phosphorylates ceramide synthase to stimulate synthesis of complex sphingolipids. *Elife* 3, pp. e.03779.

Murley, A., Yamada, J., Niles, B. J., Toulmay, A., Prinz, W. A., Powers, T. and Nunnari, J. (2017) 'Sterol transporters at membrane contact sites regulate TORC1 and TORC2 signaling', *J Cell Biol,* 216(9), pp. 2679-2689.

Roelants, F. M., Breslow, D. K., Muir, A., Weissman, J. S. and Thorner, J. (2011) Protein kinase Ypk1 phosphorylates regulatory proteins Orm1 and Orm2 to control sphingolipid homeostasis in Saccharomyces cerevisiae. *Proc. Natl. Acad. Sci. USA* 108, pp. 19222-19227.

Wang, Y., Jiang, F., Zhuo, Z., Wu, X.-H., and Wu, Y.-D. (2013) A method for WD40 repeat detection and secondary structure prediction. *PLoS One* 8, pp. e65705.

Wang, Y., Hu, X.-J., Zou, X.-D., Wu, X.-H., Ye, Z.Q., and Wu, Y.-D. (2015) WDSPdb: a database for WD40-repeat proteins. *Nucleic Acids Res*. 43, pp. D339-D344.
